# Predictive Value of Respiratory Parameters for Non-invasive Ventilation Failure in Acute Respiratory Failure: A Systematic Review

**DOI:** 10.7759/cureus.114823

**Published:** 2026-08-19

**Authors:** Mohammed Alfatih Mohammed Ramadan, Limis Mohamed Elamin Sabier Ali, Abubaker Mohammed Mohammed Elamin, Elaf Mohamedahmed Mohamed Ibrahim, Islam Suliman Abdalkareem Abaker, Osama Abdalla Sidahmed Osman, Eltayeb Osman Elfaki Omer

**Affiliations:** 1 General Medicine, Bedfordshire Hospitals NHS Trust, Bedfordshire, GBR; 2 Internal Medicine, University of Khartoum, Khartoum, SDN; 3 Internal Medicine, Ministry of Health, Shaqra, SAU; 4 Anaesthesia and Intensive Care Unit, Alnaw Teaching Hospital, Omdurman, SDN; 5 Anaesthesia, Alnaw Teaching Hospital, Omdurman, SDN; 6 Nephrology, Midland Regional Hospital Tullamore, Tullamore, IRL; 7 Endocrinology, Dr. Sulaiman Al Habib Hospital, Riyadh, SAU

**Keywords:** acute respiratory failure, endotracheal intubation, hacor score, inspiratory effort, non-invasive ventilation, pao2/fio2 ratio, prognostic factors, rox index, systematic review, treatment failure

## Abstract

Non-invasive ventilation (NIV) is first-line therapy in several forms of acute respiratory failure (ARF), but failure is common and delayed intubation carries excess mortality. The best bedside predictors, and when to measure them, are unclear. Following PRISMA 2020, four databases were searched up to 25 June 2026. Peer-reviewed primary studies reporting an association between a respiratory parameter and NIV failure in adults with ARF were eligible. Risk of bias was assessed using the Quality in Prognosis Studies (QUIPS) tool, and a narrative synthesis was performed. Study selection is presented in a PRISMA flow diagram. Twenty studies from 10 countries (~11,700 patient-episodes) covered chronic obstructive pulmonary disease (COPD) exacerbation, de novo hypoxemic failure, pneumonia and acute respiratory distress syndrome (ARDS), including approximately 8,700 hypoxemic and 3,000 hypercapnic episodes. Failure ranged from 13% to 70%, indicating marked heterogeneity. Values before NIV discriminated poorly; those at one to two hours performed well. A partial pressure of arterial oxygen to fraction of inspired oxygen ratio (PaO_2_/FiO_2_) below 146 mmHg at one hour predicted failure (odds ratio 2.51, 95% CI 1.45-4.35), while at or below 200 mmHg, the adjusted odds ratio was 4.26 (95% CI 1.62-11.16). Respiratory rate alone lost predictive value under NIV, whereas high expired tidal volume (>9-9.5 mL/kg) and unrelieved inspiratory effort marked high risk. The composite heart rate, acidosis, consciousness, oxygenation, and respiratory rate (HACOR) score reached areas under the curve of 0.85 (95% CI 0.84-0.87) to 0.94 at one to two hours, falling to 0.71-0.78 externally. Eleven studies were at low risk of bias, seven moderate, and two high. Respiratory parameters predict NIV failure most accurately when reassessed one to two hours after initiation, and composite indices such as HACOR outperform single variables. As the evidence is observational and prone to incorporation bias, confidence is moderate.

## Introduction and background

Acute respiratory failure (ARF) is among the commonest reasons for admission to intensive care and remains a leading contributor to in-hospital mortality worldwide. It is divided into two physiological types: type I, or hypoxaemic failure, in which oxygenation is the primary problem, as in pneumonia and acute respiratory distress syndrome (ARDS); and type II, or hypercapnic failure, in which the lungs cannot clear carbon dioxide adequately, as in exacerbations of chronic obstructive pulmonary disease (COPD). Non-invasive ventilation (NIV), the delivery of ventilatory support through a mask rather than a tracheal tube, has over three decades moved from an experimental technique to a first-line strategy in defined populations, principally acute hypercapnic COPD exacerbations and acute cardiogenic pulmonary oedema, where randomised evidence demonstrates reductions in intubation rate and mortality [1]. Joint European Respiratory Society and American Thoracic Society guidance formalised these indications while explicitly declining to recommend NIV for de novo hypoxaemic ARF, in which the balance of benefit and harm remains unresolved [2]. Meta-analytic evidence subsequently suggested that non-invasive oxygenation strategies as a class are associated with lower mortality than conventional oxygen in acute hypoxaemic ARF, but with substantial uncertainty about which patients benefit and for how long a trial should continue [3]. Practice has nevertheless outrun the evidence: in a large international observational study of ARDS, NIV was applied as first-line support in roughly one in six patients, and was used as often in severe as in mild disease [4].

The central clinical problem is that NIV frequently fails, and that failure is not prognostically neutral. Patients who require intubation after a period of NIV have consistently worse outcomes than patients intubated at the outset, an association observed across case-mix and severity strata and only partially explained by baseline illness severity [5]. The consequences extend beyond mortality alone. Failed NIV is associated with longer intensive care and hospital stay, with greater exposure to sedation and to invasive ventilation and its attendant complications, including ventilator-associated pneumonia and ventilator-induced lung injury, and with correspondingly greater consumption of critical care resources. Accurate early prediction therefore carries organisational as well as clinical value. Failure is also heterogeneous in its timing and mechanism: immediate failure within minutes reflects intolerance, secretion burden or agitation; early failure within the first 48 hours reflects an unresolved gas-exchange or mechanical derangement; and late failure after 48 hours reflects nosocomial complications and deconditioning, each with different remedies and different prognostic weight [6]. Superimposed on these is the concept of patient self-inflicted lung injury, whereby vigorous spontaneous breathing effort during assisted ventilation generates high distending pressures and large breath volumes that may worsen the very lung injury NIV was intended to support. This provides a mechanistic rationale for measuring the act of breathing itself, rather than only its effect on blood oxygen levels [7]. The practical corollary is that clinicians need a defensible, rapidly available way of deciding when a trial of NIV has become a delay to intubation.

Respiratory parameters are the leading candidates for this task because they are measured continuously at the bedside and are physiologically proximate to the process being monitored. Candidate variables span oxygenation, expressed as the ratio of arterial oxygen tension to inspired oxygen fraction (PaO_2_/FiO_2_) or its non-invasive equivalent using pulse oximetry (SpO_2_/FiO_2_); ventilatory drive and pattern (respiratory rate, tidal volume, minute ventilation); acid-base and gas-exchange status (pH, PaCO_2_); breathing effort, measured as the swing in oesophageal pressure during each breath; and composite indices that combine several of these into a single score. Two composite tools dominate current practice and illustrate why systematic evaluation is needed. The HACOR score, combining heart rate, acidosis, consciousness, oxygenation and respiratory rate, was developed specifically for NIV and has since been modified and externally validated in different populations with materially different reported accuracy. The respiratory rate-oxygenation (ROX) index, the ratio of SpO_2_/FiO_2_ to respiratory rate, was developed for high-flow nasal oxygen, where it discriminates success from failure and improves in accuracy as it is repeated over the first 12 hours [8,9]; its transferability to NIV is uncertain. Whether an equivalent validated, time-dependent framework exists for NIV is therefore unclear, as individual studies report divergent thresholds, measurement times and effect sizes. This systematic review was undertaken to identify all eligible primary studies evaluating respiratory parameters as predictors of NIV failure in adults with ARF of any aetiology, encompassing hypercapnic, de novo hypoxaemic, mixed, post-operative and immunocompromise-related failure; to summarise their reported predictive performance and thresholds; to determine at which time points prediction is most accurate; and to appraise the methodological quality and remaining evidence gaps of this literature.

## Review

Materials and methods

Reporting

This review was conducted and reported in accordance with the PRISMA 2020 statement [10]. The sections that follow describe, in order, the eligibility criteria, the search strategy, the study selection and duplicate screening process, data extraction and reviewer independence, resolution of disagreements, risk-of-bias assessment, the approach to evidence synthesis and heterogeneity, and the assessment of certainty. Ethical approval was not required, as only published aggregate data were analysed.

Eligibility Criteria

Eligible studies were peer-reviewed primary research, comprising prospective or retrospective cohorts and pre-specified secondary or post hoc analyses of cohorts or randomised trials, that enrolled adults treated with NIV for ARF of any aetiology and reported a quantitative association between at least one respiratory parameter and NIV failure. Respiratory parameters comprised respiratory rate, oxygenation indices (PaO_2_/FiO_2_, SpO_2_/FiO_2_ and the ROX index), pH and PaCO_2_, tidal volume, inspiratory effort measured as oesophageal pressure swing, and composite scores containing respiratory items, by which we mean instruments such as the HACOR score, its updated and modified versions, the ROX index and comparable multivariable bedside risk charts. NIV failure was accepted as defined by the study authors, most often endotracheal intubation alone or intubation or death during NIV; because these definitions are not equivalent, findings were examined separately according to which definition each study applied, and this is reported in the synthesis. Reviews, meta-analyses, conference abstracts, editorials, letters, protocols, pre-prints, case reports and series of fewer than 20 patients were excluded, as were paediatric studies, post-extubation or weaning indications, and studies of high-flow nasal oxygen without an NIV arm. The threshold of 20 patients was chosen pragmatically rather than from an established rule: with fewer than 20 participants and a typical failure rate of 30-50%, a study yields fewer than 10 outcome events, which is below the widely used minimum of 10 events per candidate predictor for a stable multivariable estimate, so smaller series could not contribute interpretable adjusted associations. No language restriction was applied; no eligible record required translation, as all articles retrieved in full text were available in English. Full criteria are given in Table 1.

**Table 1:** Eligibility criteria (PICOS framework) ARF: acute respiratory failure; AUROC: area under the receiver operating characteristic curve; CI: confidence interval; CPAP: continuous positive airway pressure; HACOR: heart rate, acidosis, consciousness, oxygenation and respiratory rate; ICU: intensive care unit; NIV: non-invasive ventilation; PEEP: positive end-expiratory pressure; ROX: ratio of SpO_2_/FiO_2_ to respiratory rate

Element	Inclusion	Exclusion
Population	Adults aged 18 years or older with ARF of any aetiology (de novo hypoxaemic, hypercapnic, acute-on-chronic, cardiogenic, post-operative or immunocompromise-related); any care setting.	Age under 18 years; chronic domiciliary ventilation or sleep-disordered breathing; NIV used solely for post-extubation prophylaxis or weaning.
Intervention/exposure	NIV (bilevel, pressure support with PEEP, or CPAP) via any interface, with at least one respiratory parameter measured before or during NIV: respiratory rate, PaO_2_/FiO_2_, SpO_2_/FiO_2_, ROX index, pH, PaCO_2_, tidal volume, inspiratory effort (oesophageal pressure swing), or a composite score containing respiratory items (e.g. HACOR, updated or modified HACOR, ROX-derived scores, multivariable bedside risk charts).	High-flow nasal oxygen or conventional oxygen without an NIV group; invasive ventilation from the outset; no respiratory parameter analysed.
Comparator	NIV success versus NIV failure; parameter values above versus below a threshold; or the same parameter at an alternative time point.	No comparison of any kind between NIV success and failure.
Outcomes	Primary: NIV failure, defined as intubation, or as intubation or death during NIV; studies were grouped by which definition was applied. Secondary: ICU, hospital, 28-day and 90-day mortality, time to intubation, AUROC, sensitivity, specificity and adjusted effect estimates with 95% CI.	Mortality reported alone without an NIV failure or intubation outcome; association not extractable.
Study design	Peer-reviewed primary research: prospective or retrospective cohorts; pre-specified secondary or post hoc analyses of cohorts or randomised trials. No language or date restriction.	Reviews, meta-analyses, conference abstracts, editorials, letters, protocols, preprints, case reports, series of fewer than 20 patients (fewer than ~10 outcome events), animal and simulation studies.

Search Strategy

PubMed, Scopus, Embase and Web of Science were searched from inception to 25 June 2026. The strategy combined controlled vocabulary (Medical Subject Headings (MeSH) in PubMed, Emtree in Embase) with free-text title, abstract and keyword terms, linked by the Boolean operators OR within each of three concept blocks (NIV; treatment failure or intubation; respiratory parameters and ARF) and AND between blocks, with truncation used for variant word endings. No database filters, language limits or date limits were applied. The full strategy for each database, with the corresponding yields, is provided in the Appendices.

Study Selection

Records were exported to reference-management software and de-duplicated. Two reviewers then screened all records independently at title and abstract, and independently assessed the full text of all potentially eligible records, with disagreements at either stage resolved by discussion and, where consensus was not reached, by adjudication from a third reviewer. Formal inter-rater agreement statistics were not calculated; this is acknowledged as a limitation.

Data Extraction

Two reviewers extracted data independently and in duplicate onto a standardised form. The form was piloted on three studies and revised before full use, which served as the calibration exercise for both reviewers. Extracted fields were: study identifiers and design; country, number of centres and recruitment period; care setting; population, case-mix and physiological type of respiratory failure; sample size and number of failures; NIV interface, mode and ventilator settings; monitoring modality; each respiratory parameter evaluated with its measurement time points and threshold; the comparator; the definition of NIV failure; predictive performance (area under the receiver operating characteristic curve, sensitivity, specificity and cut-off values); adjusted effect estimates with 95% confidence intervals and the covariates in each model; and mortality endpoints and time to intubation. Discrepancies between reviewers were resolved by returning to the source article and, where they persisted, by consensus with a third reviewer. Authors of primary studies were not contacted; fields not reported in a published article were recorded as not reported and were never imputed or estimated.

Handling of Overlapping Cohorts

Because several research groups have published repeatedly from the same prospectively maintained registries, potential cohort overlap was assessed systematically. Studies were compared on recruiting centre, city, recruitment dates, eligibility criteria and sample size, and author lists were examined for shared investigators. Where overlap was suspected, full texts were re-read, and earlier reports consulted to establish the recruitment window. Studies sharing a source registry were retained as separate reports because each addressed a distinct parameter, population or validation question, but were flagged as non-independent, counted only once in statements about the weight of evidence, and identified explicitly in the Results and Discussion. This is also the principal reason quantitative pooling was not attempted, since double-counting shared patients would produce spuriously narrow confidence intervals.

Risk of Bias

Risk of bias was assessed with the Quality In Prognosis Studies (QUIPS) tool across its six domains by two independent reviewers [11]. Before formal assessment, both reviewers studied the QUIPS guidance and independently applied the six domain judgements to three studies, comparing and reconciling their ratings in order to standardise interpretation of each domain; the prompts used for each domain were then fixed and applied uniformly to all 20 studies. Disagreements were resolved by consensus, and formal agreement statistics were not calculated. Particular attention was paid to incorporation bias, arising where the parameter under evaluation also contributed to the clinical decision to intubate.

Data Synthesis and Assessment of Heterogeneity

Meta-analysis was not undertaken. Heterogeneity was appraised in three domains before this decision was taken. Clinical heterogeneity was substantial: studies differed in physiological type of respiratory failure, aetiology, illness severity, NIV protocols and thresholds for intubation, and in care setting. Methodological heterogeneity was equally marked: designs ranged from retrospective cohorts to post hoc analyses of randomised trials; the same parameter was assessed at time points ranging from before NIV to 48 hours; thresholds for a given parameter differed between cohorts; the definition of failure varied between intubation alone and intubation or death during NIV; and effect metrics were mixed, with some studies reporting discrimination and others reporting adjusted association measures from differently specified models. Statistical heterogeneity could not be quantified, because these differences, together with overlap between several large cohorts, made a pooled estimate uninterpretable rather than merely imprecise. A structured narrative synthesis was therefore performed, grouping studies by parameter domain and examining findings by physiological type, ARDS severity category, care setting and measurement time point. Because no meta-analysis was performed, formal assessment of publication bias by funnel plot or regression-based asymmetry testing was not possible; the potential for small-study and positive-result reporting bias is therefore acknowledged but unquantified.

Certainty of Evidence

The certainty of the evidence for each parameter domain was rated as high, moderate, low or very low using an adaptation of the Grading of Recommendations Assessment, Development and Evaluation (GRADE) approach for prognostic factor reviews, considering risk of bias, consistency in the direction and magnitude of association, precision, directness to the clinical decision to intubate, and the number of independent cohorts. Ratings were assigned by consensus between two reviewers.

Results

Study Selection

**Figure 1:**
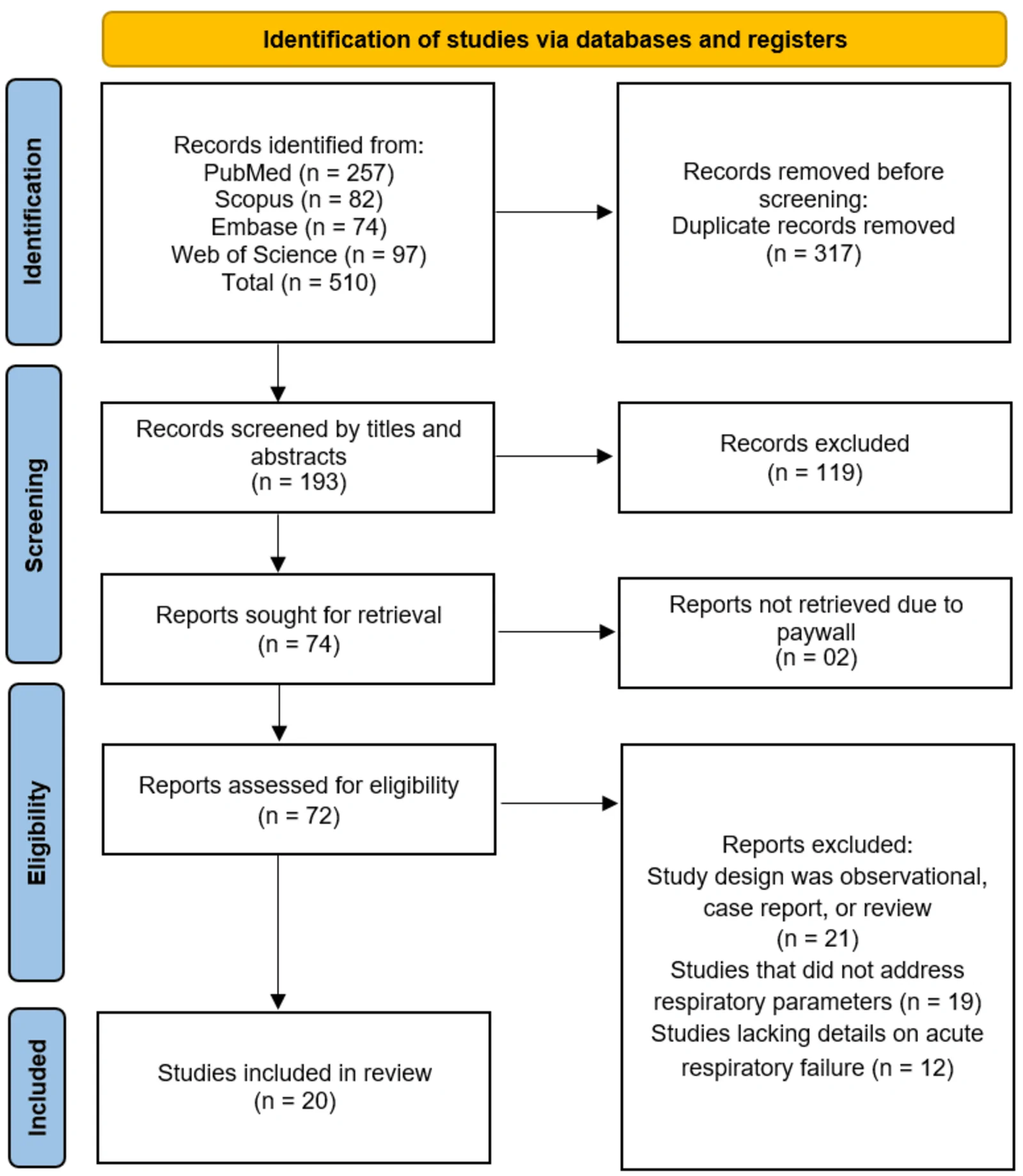
PRISMA flowchart of the study selection process

The search returned 510 records: 257 from PubMed, 97 from Web of Science, 82 from Scopus and 74 from Embase. After removal of 317 duplicates, 193 records were screened by title and abstract, and 119 were excluded. Of the 74 reports sought for retrieval, two could not be obtained because of paywall restrictions, leaving 72 assessed at full text. Fifty-two were excluded: 21 for ineligible publication type, 19 that did not address respiratory parameters, and 12 that lacked adequate detail on ARF. Twenty studies met all eligibility criteria and were included in the narrative synthesis [12-31]. The selection process is summarised in Figure 1.

Across these 20 studies, oxygenation indices were evaluated in eight [14,17-19,22,23,25,31], respiratory rate in seven [15,21,23,24,28,30,31], acid-base indices (pH or PaCO_2_) in five [12,13,15,16,25], tidal volume in two [20,23], directly measured inspiratory effort in one [27], and composite bedside scores in seven [15,21,24,26,28-30], of which six evaluated the HACOR score specifically [21,24,26,28-30]. Several studies contributed to more than one domain.

Study Characteristics

The characteristics of the 20 included studies are presented in Table 2. Publication dates spanned 27 years, from 1995 [12] to 2022 [29-31], and the studies were conducted across 10 countries: Italy [12,13,15,27], a multinational European and United States consortium [14], Singapore [16], the United States [17], France [18,20,23], India [19], China [21,24,25,28,29], an international consortium spanning 50 countries [22], Spain [26], and China with Turkey [30,31]. Fourteen studies used prospective or consecutive observational cohort designs, two were retrospective cohorts [12,26], and four were pre-specified secondary or post hoc analyses of prospectively collected cohorts or of a randomised controlled trial [22,23,29,31]. Sample sizes ranged from 30 patients in a physiological pilot study [27] to 2,749 NIV episodes in 2,711 consecutive patients [26].

**Table 2:** Characteristics of the 20 included studies Type I: hypoxaemic respiratory failure; Type II: hypercapnic or acute-on-chronic respiratory failure. APACHE II: Acute Physiology and Chronic Health Evaluation II; aOR: adjusted odds ratio; ARDS: acute respiratory distress syndrome; ARF: acute respiratory failure; AUROC: area under the receiver operating characteristic curve; COPD: chronic obstructive pulmonary disease; GCS: Glasgow Coma Scale; HACOR: heart rate, acidosis, consciousness, oxygenation and respiratory rate; ICU: intensive care unit; NIV: non-invasive ventilation; OR: odds ratio; PBW: predicted body weight; RR: respiratory rate; ROX: ratio of SpO_2_/FiO_2_ to respiratory rate; Vt/Vte: tidal volume/expired tidal volume

First author, year [ref]	Country	Study design	Population and setting	Sample size	Exposure (respiratory parameter)	Type of ARF	Comparator	Outcome	Key findings
Ambrosino, 1995 [12]	Italy	Retrospective cohort, single centre	COPD with ARF; respiratory ICU	59 episodes in 47 patients	Baseline pH, PaCO_2_, PaO_2_/FiO_2_, respiratory rate	Type II	NIV success vs failure	Intubation or death	Failure 22%; simple baseline physiological and nutritional variables discriminated success from failure.
Moretti, 2000 [13]	Italy	Prospective cohort, two respiratory ICUs	COPD with hypercapnic failure who initially responded to NIV	137	Admission pH and PaCO_2_; response to NIV	Type II	Sustained success vs late failure	Late NIV failure (>48 h); mortality	Late failure 23% after a mean of 8.4 days; associated with lower admission pH and medical complications.
Antonelli, 2001 [14]	Europe and USA	Prospective multicentre cohort, eight ICUs	Hypoxaemic ARF of mixed aetiology; ICU	354	PaO_2_/FiO_2_ at baseline and 1 h	Type I	PaO_2_/FiO_2_ >146 vs <=146 mmHg at 1 h	Endotracheal intubation	Failure 30%; PaO_2_/FiO_2_ <=146 mmHg at 1 h independently predicted failure (OR 2.51).
Confalonieri, 2005 [15]	Italy	Prospective multicentre cohort, 13 units	COPD exacerbation; ICU, intermediate care units and wards	1,033	Respiratory rate and pH (with GCS and APACHE II) at admission and 2 h	Type II	Risk-chart strata	NIV failure (intubation or death)	Failure 23%; risk charts derived at admission and 2 h, with low pH the dominant determinant of risk.
Phua, 2005 [16]	Singapore	Prospective cohort, single medical ICU	Hypercapnic failure: COPD (n=43) vs other conditions (n=68)	111	PaCO_2_ and pH at baseline and 1 h; respiratory rate	Type II	COPD vs non-COPD; success vs failure	Endotracheal intubation	Failure 19% in COPD vs 47% in non-COPD; PaCO_2_ at 1 h predicted failure (OR 1.22 per 5 mmHg).
Rana, 2006 [17]	USA	Observational cohort, two ICUs	Acute lung injury treated with NIV as initial mode; ICU	54	PaO_2_/FiO_2_ and NIV respiratory parameters; base deficit	Type I	Success vs failure; per-unit change	Intubation; hospital mortality	Failure 70%; metabolic acidosis and severe hypoxaemia predicted failure; all 19 patients with shock failed.
Thille, 2013 [18]	France	Prospective observational cohort, single ICU	Non-hypercapnic hypoxaemic failure; ARDS (n=82), non-ARDS (n=31)	113	PaO_2_/FiO_2_ at initiation; ARDS severity category	Type I	PaO_2_/FiO_2_ >150 vs <=150 mmHg	Intubation; ICU mortality	Intubation 31%, 62% and 84% in mild, moderate and severe ARDS; PaO_2_/FiO_2_ >150 mmHg favourable.
Chawla, 2016 [19]	India	Prospective observational cohort, single centre	ARDS by Berlin definition; ICU	96	Baseline PaO_2_/FiO_2_; ARDS severity	Type I	NIV success vs failure	NIV failure; ICU mortality	Failure 43.8%; low baseline PaO_2_/FiO_2_, shock and ARDS severity associated with failure.
Carteaux, 2016 [20]	France	Prospective observational cohort, single ICU	De novo acute hypoxaemic respiratory failure; ICU	62	Mean expired tidal volume (mL/kg PBW) during NIV	Type I	Vte <=9.5 vs >9.5 mL/kg PBW	NIV failure (intubation)	Vte >9.5 mL/kg PBW predicted failure; a lung-protective Vte was unachievable in every patient.
Duan, 2017 [21]	China	Prospective cohort (derivation and validation)	Hypoxaemic ARF; respiratory ICU	449 + 358	HACOR score at 0, 1, 12, 24 and 48 h	Type I	HACOR <=5 vs >5 points	NIV failure (intubation or death)	Failure 47.8% and 39.4%; HACOR at 1 h achieved AUROC 0.88 with a cut-off of 5 points.
Bellani, 2017 [22]	50 countries	Secondary analysis of a prospective international cohort	ARDS managed with NIV on days 1-2; ICU	436	PaO_2_/FiO_2_ (Berlin severity category)	Type I	Mild vs moderate vs severe ARDS	NIV failure; ICU and hospital mortality	Failure 22.2%, 42.3% and 47.1% by severity; hospital mortality 45.4% after failure vs 16.1% after success.
Frat, 2018 [23]	France and Belgium	Post hoc analysis of a multicentre randomised trial	De novo acute hypoxaemic respiratory failure; ICU	310	Respiratory rate, PaO_2_/FiO_2_ and tidal volume at baseline and 1 h	Type I	RR >=30 vs <30/min; PaO_2_/FiO_2_ <=200 mmHg; Vt >9 mL/kg PBW	Intubation; 90-day mortality	At 1 h, PaO_2_/FiO_2_ <=200 mmHg (aOR 4.26) and Vt >9 mL/kg PBW (aOR 3.14) predicted intubation.
Duan, 2019 [24]	China	Prospective cohort with internal and external validation	COPD exacerbation with ARF; respiratory ICU	500 + 323 + 395	HACOR score at 1-2 h	Type II	HACOR <=5 vs >5 points	NIV failure (intubation or death)	AUROC 0.90, 0.89 and 0.71; failure 50.2% when score >5, where early intubation lowered mortality.
Chen, 2020 [25]	China	Prospective cohort, single centre	COPD exacerbation receiving NIV beyond 48 h; respiratory ICU	291	pH, PaO_2_/FiO_2_, respiratory rate and heart rate at 0 and 1-2 h	Type II	Sustained success vs late failure	Late NIV failure; hospital mortality	Late failure 16%; pH at 1-2 h independently predicted failure (OR 2.06 per 0.05 decrease).
Carrillo, 2020 [26]	Spain	Retrospective cohort, single polyvalent ICU	Hypoxaemic ARF of mixed aetiology; ICU	2,749 episodes in 2,711 patients	HACOR score before NIV and at 1, 6, 12, 24 and 48 h	Type I	HACOR strata; success vs failure	NIV failure; mortality by timing of failure	Failure 35%; AUROC 0.88 at 1 h and above 0.90 in pneumonia and ARDS, but no difference before NIV.
Tonelli, 2020 [27]	Italy	Prospective physiological pilot cohort, single centre	De novo hypoxaemic failure undergoing a 24-h NIV trial; ICU	30	Oesophageal and transpulmonary pressure swing, tidal volume, respiratory rate	Type I	Fall in oesophageal pressure swing >=10 vs <10 cmH_2_O at 2 h	Intubation within 24 h	A fall of >=10 cmH_2_O at 2 h predicted success (OR 15; AUROC 0.97), but from only 30 patients.
Ding, 2021 [28]	China	Prospective cohort, single respiratory ICU	Non-COPD acute-on-chronic failure (pH <7.35, PaCO_2_ >45 mmHg)	148	HACOR score at 0, 1-2, 12 and 24 h	Type II	HACOR strata; success vs failure	NIV failure (intubation or death)	Failure 13%; AUROC rose from 0.69 at initiation to 0.94 at 24 h as cut-offs fell from 7 to 2 points.
Bai, 2022 [29]	China	Secondary analysis of two prospective databases	Moderate-to-severe ARDS (PaO_2_/FiO_2_ <=150 mmHg) on first-line NIV	224	Change in HACOR score after 1-2 h	Type I	Responders (fall >=1 point) vs non-responders	NIV failure; 28-day mortality; time to intubation	Failure 56%; a fall of >=1 point halved failure (36% vs 72%) and reduced 28-day mortality.
Duan, 2022 [30]	China and Turkey	Prospective multicentre observational cohort, 18 hospitals	Hypoxaemic respiratory failure receiving NIV; ICU	1,451 + 728	Updated HACOR score (original items plus 6 baseline variables) at 1-2, 12 and 24 h	Type I	Updated vs original HACOR; score strata	NIV failure	Updated score achieved AUROC 0.85 (training) and 0.78 (validation); 4 of 6 added items are non-respiratory.
Duan, 2022 [31]	China and Turkey	Secondary analysis of a prospective multicentre study	De novo ARF, hypercapnic patients excluded; ICU	1,286	ROX index before NIV and at 1-2, 12 and 24 h	Type I	ROX strata; ROX vs PaO_2_/FiO_2_	NIV failure	Failure 44%; AUROC rose from 0.64 before NIV to 0.77 at 24 h, no better than PaO_2_/FiO_2_ alone.

By physiological type, 13 studies enrolled patients with type I hypoxaemic failure, including de novo hypoxaemic failure, acute lung injury and ARDS, contributing approximately 8,700 patient-episodes (74% of the evidence base) [14,17-23,26,27,29-31]; and seven studies enrolled patients with type II hypercapnic or acute-on-chronic failure, predominantly COPD exacerbations, contributing approximately 3,000 patient-episodes (26%) [12,13,15,16,24,25,28]. No included study reported a mixed cohort in which type I and type II patients were analysed together without separation. The aggregate figure of approximately 11,700 patient-episodes overstates the number of unique individuals, because seven studies drew on overlapping prospectively maintained registries in Chongqing, China [21,24,25,28-31]. Only one duplication can be quantified from the published reports: the 1,286 patients analysed in [31] are drawn from the multicentre cohort of 2,179 reported in [30], and removing this single documented overlap reduces the total to approximately 10,400 patient-episodes. A further secondary analysis of two previously reported databases [29] almost certainly shares patients with [21] and [30], but recruitment windows and centre identifiers are not reported in sufficient detail to quantify this, so the true number of unique individuals is lower than 10,400 and cannot be determined precisely. All seven Chongqing reports are treated as non-independent throughout.

Baseline demographic and severity data were extracted where available but could not be aggregated. Age and sex distribution were reported in differing summary formats across cohorts, and illness severity was quantified with three different instruments - the Simplified Acute Physiology Score II [14], the Acute Physiology and Chronic Health Evaluation II score [15,16,25] and the Sequential Organ Failure Assessment score [30] - so no common severity metric exists across the evidence base. A pooled description of age, sex or severity would therefore be misleading, and readers should judge applicability from the population and setting columns of Table 2 rather than from a summary statistic.

Care setting was overwhelmingly the intensive care or respiratory intensive care unit, which applied to 19 of the 20 studies. Only one national multicentre study extended recruitment beyond critical care, including respiratory intermediate care units and general wards [15]. No included study was conducted in an emergency department or a respiratory high-dependency unit as its primary setting. This is an important constraint on applicability, because NIV is increasingly initiated outside the intensive care unit, where monitoring intensity, staffing ratios and the speed with which intubation can be delivered all differ; the thresholds reported here were derived under close critical-care observation and should not be assumed to transfer unchanged to ward or emergency-department practice. Reported NIV failure ranged from 13% in non-COPD acute-on-chronic hypercapnic failure [28] and 19% in COPD [16] to 56% in moderate-to-severe ARDS [29] and 70% in acute lung injury [17]. This more than fivefold range should be read as an index of heterogeneity rather than of biological variation alone, since it reflects differences in ARF aetiology, NIV protocols and interfaces, the timing at which parameters were measured, local criteria for intubation and the definition of failure applied, and the balance of intensive care versus ward settings.

NIV Delivery and Monitoring

Ventilatory support was delivered by oronasal or full-face mask in every included study; no study used a helmet interface, and no study reported nasal-mask delivery as its primary modality. The mode was pressure support with positive end-expiratory pressure (PEEP) in the majority of cohorts, with continuous positive airway pressure used in a minority of hypoxaemic cohorts. Ventilator settings were individually titrated rather than fixed by protocol in all studies that described them: inspiratory pressure or pressure support was adjusted to achieve an acceptable tidal volume, patient comfort and a reduction in respiratory rate, expiratory pressure or PEEP was titrated against oxygenation, and FiO_2_ was titrated to a target oxygen saturation, most commonly at or above 90%. Reporting of these settings was, however, inconsistent and incomplete: no included study reported a mean FiO_2_, inspiratory positive airway pressure or expiratory positive airway pressure in a form that would permit aggregation across cohorts, and several reported settings only as titration targets. Pooled values for these variables therefore cannot be derived from the published literature and are not presented here. Monitoring was by intermittent arterial blood gas analysis at protocol-specified time points in every included study; none reported routine continuous capnographic monitoring, and none used end-tidal carbon dioxide as a predictor of failure.

Oxygenation

Oxygenation indices were the most frequently evaluated parameter domain and the most consistent predictors of failure, provided they were measured after NIV had been started rather than at baseline alone (Table 3). In the earliest large multicentre cohort, 354 patients with hypoxaemic ARF had a failure rate of 30%, and a PaO_2_/FiO_2_ ratio of 146 mmHg or less after one hour of NIV was independently associated with failure (odds ratio 2.51, 95% CI 1.45-4.35), alongside a Simplified Acute Physiology Score II of 35 or more and a diagnosis of ARDS or community-acquired pneumonia [14]. A comparable relationship was demonstrated in acute lung injury, where each unit decrease in PaO_2_/FiO_2_ increased the odds of failure by 3% (odds ratio 1.03, 95% CI 1.01-1.05) among patients without shock [17].

**Table 3:** Reported predictive performance of respiratory parameters for NIV failure aOR: adjusted odds ratio; APACHE II: Acute Physiology and Chronic Health Evaluation II; ARDS: acute respiratory distress syndrome; AUROC: area under the receiver operating characteristic curve; CI: confidence interval; GCS: Glasgow Coma Scale; HACOR: heart rate, acidosis, consciousness, oxygenation and respiratory rate; NIV: non-invasive ventilation; OR: odds ratio; PBW: predicted body weight; RR: respiratory rate; ROX: ratio of SpO_2_/FiO_2_ to respiratory rate

Study	Respiratory parameter	Timing	Threshold/cut-off	Discrimination	Association with NIV failure (95% CI)
Antonelli, 2001 [14]	PaO_2_/FiO_2_	1 h of NIV	<=146 mmHg	Not reported	OR 2.51 (1.45-4.35)
Confalonieri, 2005 [15]	Composite risk chart (RR, pH, GCS, APACHE II)	Admission and 2 h	Predicted risk >50%	Sensitivity 33%, specificity 96.7% (admission); 52.9% and 94.1% (2 h)	Graded risk strata; low pH the dominant determinant
Phua, 2005 [16]	PaCO_2_	1 h of NIV	Per 5 mmHg increase	Not reported	OR 1.22 per 5 mmHg (non-COPD)
Rana, 2006 [17]	PaO_2_/FiO_2_	At initiation	Per unit decrease	Not reported	OR 1.03 (1.01-1.05) per unit fall
Thille, 2013 [18]	PaO_2_/FiO_2_	At initiation	>150 vs <=150 mmHg (moderate ARDS)	Not reported	Failure 45% vs 74% (p=0.04)
Carteaux, 2016 [20]	Expired tidal volume	Mean over NIV duration	>9.5 mL/kg PBW	Reported as accurate in moderate-to-severe hypoxaemia; AUROC not reported	Independently associated with failure
Duan, 2017 [21]	HACOR score	1 h of NIV	5 points	AUROC 0.88	Higher score, higher failure probability
Bellani, 2017 [22]	PaO_2_/FiO_2_ (Berlin category)	Days 1-2	Mild/moderate/severe	Not reported	Failure 22.2%/42.3%/47.1%
Frat, 2018 [23]	Respiratory rate	Baseline	>=30 breaths/min	Not reported for RR alone	OR 2.76 (1.13-6.75) under standard oxygen; not predictive under NIV
Frat, 2018 [23]	PaO_2_/FiO_2_	1 h of NIV	<=200 mmHg	Not reported	aOR 4.26 (1.62-11.16)
Frat, 2018 [23]	Tidal volume	1 h of NIV	>9 mL/kg PBW	Not reported	aOR 3.14 (1.22-8.06); also predicted 90-day mortality
Duan, 2019 [24]	HACOR score	1-2 h of NIV	>5 points	AUROC 0.90/0.89/0.71 (derivation/internal/external)	Failure 50.2% when score >5
Chen, 2020 [25]	pH	1-2 h of NIV	Per 0.05 decrease below 7.35	Not reported	OR 2.06 (1.41-3.00) for late failure
Chen, 2020 [25]	PaO_2_/FiO_2_	1-2 h of NIV	Per unit increase	Not reported	OR 0.992 (0.986-0.998), protective
Carrillo, 2020 [26]	HACOR score	Before NIV and at 1, 6, 12, 24, 48 h	Not fixed	AUROC 0.88 at 1 h; >0.90 in pneumonia and ARDS	No difference before NIV; clear separation from 1 h onward
Tonelli, 2020 [27]	Fall in oesophageal pressure swing	2 h of NIV	>=10 cmH_2_O fall	AUROC 0.97 (0.91-1.00); n=30, 12 events, not externally validated	OR 15 (2.8-110) for avoiding intubation; wide CI reflects small sample
Ding, 2021 [28]	HACOR score	0, 1-2, 12 and 24 h	7/5/4/2 points	AUROC 0.69/0.91/0.91/0.94	Sensitivity 90%, specificity 85% at 1-2 h
Bai, 2022 [29]	Change in HACOR score	1-2 h of NIV	Fall of >=1 point	Not reported	Failure 36% vs 72% (p<0.01); 28-day mortality 32% vs 47% (p=0.04)
Duan, 2022 [30]	Updated HACOR score	1-2, 12 and 24 h	7/10.5/14 points (25%/50%/75% risk)	AUROC 0.85 (0.84-0.87) training; 0.78 (0.75-0.81) validation	Failure 12.4%, 38.2%, 67.1% and 83.7% across strata
Duan, 2022 [31]	ROX index	Before NIV and 1-2, 12, 24 h	Strata <=2 to >10	AUROC 0.64 (0.61-0.67) before NIV; 0.71 (0.68-0.74), 0.74 (0.71-0.77), 0.77 (0.74-0.80)	Failure 92.3% (ROX <=2) to 29.5% (ROX >10); not superior to PaO_2_/FiO_2_

Severity of hypoxaemia at presentation stratified risk in a graded fashion. In a French cohort of 113 patients, intubation rates rose from 31% in mild to 62% in moderate and 84% in severe ARDS, and among patients with moderate ARDS a PaO_2_/FiO_2_ above 150 mmHg was associated with a lower failure rate (45% versus 74%, p = 0.04) [18]. The same gradient was reproduced internationally in the LUNG SAFE sub-study, in which failure occurred in 22.2%, 42.3% and 47.1% of patients with mild, moderate and severe ARDS, respectively, and hospital mortality was 16.1% after success compared with 45.4% after failure [22]. An Indian cohort of 96 ARDS patients reported failure in 43.8% and identified a low baseline PaO_2_/FiO_2_, shock and ARDS severity as associated factors [19].

The predictive value of oxygenation measured one hour after initiation was quantified most precisely in a post hoc analysis of a multicentre randomised trial: among patients receiving NIV, a PaO_2_/FiO_2_ of 200 mmHg or less at one hour was an independent predictor of intubation (adjusted odds ratio 4.26, 95% CI 1.62-11.16) [23]. The ROX index, originally developed for high-flow nasal oxygen, was tested in 1,286 patients with de novo ARF receiving NIV; failure rates fell monotonically across pre-treatment strata from 92.3% at values of 2 or less to 29.5% above 10, and discrimination improved with time, from an area under the curve of 0.64 (95% CI 0.61-0.67) before treatment to 0.71 (95% CI 0.68-0.74), 0.74 (95% CI 0.71-0.77) and 0.77 (95% CI 0.74-0.80) at one to two, 12 and 24 hours [31].

Taken together, the relative performance of the oxygenation indices can be summarised as follows, and is tabulated by domain in Table 4. Baseline PaO_2_/FiO_2_ offers only limited discrimination but does stratify risk usefully into broad severity bands, as the ARDS gradient shows. PaO_2_/FiO_2_ measured at one hour is the strongest single oxygenation predictor, with adjusted odds ratios between 2.51 and 4.26 across independent cohorts. The ROX index performs moderately and improves with repetition, but in the one study that compared them directly, it did not outperform PaO_2_/FiO_2_ alone at any time point [31]. Three practical implications follow. First, the increment in discrimination that ROX offers over PaO_2_/FiO_2_ under NIV was not statistically or clinically meaningful in that comparison, so ROX cannot currently be recommended as a superior alternative in ventilated patients. Second, where an arterial blood gas is already being obtained, PaO_2_/FiO_2_ remains the simpler and better-supported choice under NIV. Third, the specific advantage of ROX is that it requires only pulse oximetry and a respiratory rate, so its likely role is in settings where arterial sampling is not routinely available or where non-invasive serial monitoring is preferred; that role has not yet been formally tested in an NIV population.

**Table 4:** Synthesis of evidence, discrimination and certainty by respiratory parameter domain Certainty was rated using an adaptation of the GRADE approach for prognostic factor reviews. Several studies contribute to more than one domain. GRADE: Grading of Recommendations Assessment, Development and Evaluation; Type II: hypercapnic or acute-on-chronic respiratory failure. AUROC: area under the receiver operating characteristic curve; HACOR: heart rate, acidosis, consciousness, oxygenation and respiratory rate; NIV: non-invasive ventilation; OR/aOR: odds ratio/adjusted odds ratio; PBW: predicted body weight; ROX: ratio of SpO_2_/FiO_2_ to respiratory rate; RR: respiratory rate; Vte: expired tidal volume

Parameter domain	Studies, n [ref]	Optimal timing	Key threshold	Typical discrimination	Consistency	Certainty (reason for downgrading)
Oxygenation (PaO_2_/FiO_2_, SpO_2_/FiO_2_, ROX)	8 [14,17-19,22,23,25,31]	1-2 h after start	PaO_2_/FiO_2_ <=146-200 mmHg at 1 h; <=150 mmHg for ARDS stratification	OR 1.03-4.26; ROX AUROC 0.64 rising to 0.77	Consistent (8 of 8)	Moderate (risk of bias: unblinded outcome, incorporation bias)
Respiratory rate	7 [15,21,23,24,28,30,31]	Baseline only, under standard oxygen	>=30 breaths/min	No AUROC reported for RR alone; OR 2.76 (standard oxygen only)	Inconsistent as a single predictor	Low (inconsistency: association reverses once NIV applied; risk of bias)
Acid-base status (pH, PaCO_2_)	5 [12,13,15,16,25]	Presentation and 1-2 h	pH <7.25-7.30, or failure to correct by 2 h	Sensitivity 33-53%, specificity 94-97% (risk chart); OR 1.22-2.06	Consistent (5 of 5) in type II failure	Moderate (risk of bias: unblinded outcome ascertainment)
Tidal volume and inspiratory effort	3 [20,23,27]	1-2 h after start	Vte >9-9.5 mL/kg PBW; oesophageal pressure swing fall <10 cmH_2_O	aOR 3.14 (tidal volume); AUROC 0.97 from a single 30-patient study	Consistent (3 of 3)	Low (imprecision and indirectness: few small cohorts, no external validation)
Composite scores (HACOR, risk charts)	7 [15,21,24,26,28-30]	1-2 h after start	HACOR >5 points; updated HACOR 7/10.5/14 points	AUROC 0.85-0.94 in derivation; 0.71-0.78 on external validation	Consistent (7 of 7)	Moderate (risk of bias and cohort overlap; optimism on external validation)

Respiratory Rate

Respiratory rate behaved differently from oxygenation and, taken alone, proved a comparatively weak discriminator once NIV had been applied. In the post hoc analysis of a randomised trial, a respiratory rate of 30 breaths per minute or more predicted intubation among patients receiving standard oxygen (odds ratio 2.76, 95% CI 1.13-6.75) but did not predict intubation among patients receiving either NIV or high-flow nasal oxygen [23]. This dissociation is clinically important, because it indicates that positive pressure can normalise respiratory frequency without correcting the underlying derangement. Consistent with this, respiratory rate contributed to prediction principally as a component of composite indices rather than as a stand-alone variable [15,21,24,28,30], and the ROX index, in which respiratory rate forms the denominator, offered no advantage over PaO_2_/FiO_2_ at most time points [31]. In hypercapnic failure, however, a persistently elevated respiratory rate at presentation retained prognostic weight and was an explicit item in the admission and two-hour risk charts derived from 1,033 consecutive COPD patients [15].

Three qualifications should be attached to these observations. The first concerns which aspect of respiratory rate is informative. The included studies almost all analysed a single absolute value at a fixed time point rather than its trajectory, so the review can say little about whether a fall in respiratory rate after NIV, persistent tachypnoea despite adequate support, or a failure of respiratory rate to decrease at all carries distinct prognostic meaning. Only the composite-score studies capture change over time, and their respiratory rate is one weighted item among five and cannot be isolated. The distinction matters clinically, because persistent tachypnoea with unchanged oxygenation and a normalising respiratory rate with unchanged oxygenation may well have different implications, and neither has been tested directly. The second concerns quantitative performance: no included study reported an area under the curve, sensitivity or specificity for respiratory rate as an isolated predictor under NIV, so it cannot be compared numerically with oxygenation or with composite scores, and Table 3 records only the association measure available. The third concerns effect modification. The predictive value of respiratory rate is likely to differ by ARF aetiology, being weak in de novo hypoxaemic failure where it was formally non-predictive under NIV [23] yet retained in hypercapnic failure at presentation [15], and it is also likely to vary with underlying disease, with the timing of assessment and with the level of pressure support applied, since higher support directly lowers respiratory frequency. None of the included studies stratified their analysis in this way, so these remain plausible sources of the observed inconsistency rather than demonstrated effect modifiers.

Acid-Base Status

In type II hypercapnic and acute-on-chronic failure, acid-base status rather than oxygenation dominated risk stratification. The earliest included study analysed 59 episodes of ARF in 47 COPD patients and reported failure in 22%, identifying simple baseline physiological and nutritional variables as correlates of success [12]. In the largest COPD dataset of 1,033 consecutive patients, two risk charts were constructed, one at admission and one after two hours of NIV, incorporating Glasgow Coma Scale score, Acute Physiology and Chronic Health Evaluation II score, respiratory rate and pH; a low pH at either time point was the dominant determinant of predicted failure risk, and the two-hour chart discriminated better than the admission chart, with sensitivity and specificity for a predicted risk above 50% of 33% and 96.7% on admission and 52.9% and 94.1% at two hours [15]. In a Singaporean cohort of 111 patients, failure was markedly lower in COPD than in non-COPD causes (19% versus 47%); in the non-COPD group, pneumonia (odds ratio 5.63), a higher Acute Physiology and Chronic Health Evaluation II score (odds ratio 2.59 per 5 points), a more rapid heart rate (odds ratio 1.22 per 5 beats per minute) and a higher PaCO_2_ one hour after NIV (odds ratio 1.22 per 5 mmHg) independently predicted failure [16].

Whether PaCO_2_ predicts failure independently of pH cannot be resolved from these data, and the distinction has clinical importance. In the Singaporean cohort, PaCO_2_ at one hour entered the multivariable model while pH did not, which is consistent with an independent effect of carbon dioxide retention [16]; but in the largest COPD dataset pH was the dominant term and PaCO_2_ was not retained [15], and the two variables are mathematically coupled through the Henderson-Hasselbalch relationship, so a model containing both is unstable. No included study formally tested mediation. The most defensible reading is that the prognostic signal in type II failure resides principally in the degree and persistence of acidaemia, that a rising or unfalling PaCO_2_ at one to two hours is a marker of the same failure to ventilate, and that clinicians should act on the pH trajectory rather than treating PaCO_2_ as a separate independent predictor.

The trajectory of acid-base variables also predicted late failure. Among 137 COPD patients who had initially responded to NIV, 31 (23%, 95% CI 18-33) subsequently deteriorated after a mean of 8.4 days, and a lower admission pH, pre-admission functional limitation and medical complications such as hyperglycaemia were associated with this late failure [13]. In a cohort of 291 COPD patients ventilated beyond 48 hours, late failure occurred in 48 patients (16%), and pH at one to two hours of NIV was an independent risk factor (odds ratio 2.06, 95% CI 1.41-3.00 per 0.05 decrease below 7.35), while a higher Glasgow Coma Scale score (odds ratio 0.50, 95% CI 0.34-0.73 per point) and a higher PaO_2_/FiO_2_ (odds ratio 0.992, 95% CI 0.986-0.998 per unit) were protective [25]. These two cohorts are the only included studies to address late failure; they were conducted two decades apart in different health systems, and neither set of predictors has been externally validated in an independent population. They agree in direction, in that lower pH and greater comorbid burden predict late deterioration, but the specific thresholds and effect sizes should be regarded as cohort-specific findings awaiting confirmation rather than as validated rules.

Two limitations qualify this domain as a whole. Methodologically, all five contributing studies were observational, none blinded the intubation decision to the acid-base values under evaluation, NIV protocols and criteria for escalation differed between them, and the definition of failure varied between intubation alone and intubation or death during NIV, so the reported associations are susceptible to confounding by indication and to incorporation bias. In terms of applicability, every one of these findings derives from hypercapnic or acute-on-chronic populations, predominantly COPD. They should not be extrapolated to de novo hypoxaemic respiratory failure, ARDS, cardiogenic pulmonary oedema or post-operative respiratory failure, in which acidaemia has a different physiological meaning and in which oxygenation and respiratory mechanics, rather than pH, carried the prognostic signal in this review.

Tidal Volume and Inspiratory Effort

Two studies examined tidal volume during NIV and one examined inspiratory effort directly, so this is the most sparsely evidenced domain in the review and its findings, although mechanistically informative, rest on the smallest number of patients. In 62 patients with de novo acute hypoxaemic ARF, a lung-protective expired tidal volume proved almost impossible to achieve, since no patient maintained a mean expired tidal volume below 6 mL/kg of predicted body weight, and a high expired tidal volume was independently associated with failure; among patients with moderate-to-severe hypoxaemia, an expired tidal volume above 9.5 mL/kg of predicted body weight predicted failure [20]. A closely similar threshold was corroborated in the post hoc trial analysis, in which a tidal volume greater than 9 mL/kg of predicted body weight one hour after initiation independently predicted intubation (adjusted odds ratio 3.14, 95% CI 1.22-8.06) and remained independently associated with 90-day mortality [23].

Inspiratory effort was measured directly in a physiological pilot study of 30 consecutive patients with de novo hypoxaemic ARF undergoing a 24-hour NIV trial [27]. All patients had a high inspiratory effort at baseline. Two hours after starting NIV, the tidal swing in oesophageal pressure had fallen markedly in the 18 patients who completed the trial successfully but remained essentially unchanged in the 12 who required intubation (median 11 cmH_2_O, interquartile range 8-15, versus 31.5 cmH_2_O, interquartile range 30-36; p < 0.0001), whereas other variables diverged only later. A reduction of 10 cmH_2_O or more at two hours was associated with avoidance of intubation, with an odds ratio of 15 (95% CI 2.8-110) and an area under the curve of 0.97 (95% CI 0.91-1.00). This is the highest discrimination reported in the review, but it should be interpreted with caution rather than as evidence of a near-perfect test. The estimate derives from a single-centre pilot study of 30 patients with only 12 events; the confidence interval around the odds ratio spans an order of magnitude, from 2.8 to 110, which is itself a direct expression of that small sample; and an area under the curve estimated in the same small sample in which the threshold was identified is optimistically biased in the absence of independent validation.

Practical constraints further limit immediate application. Oesophageal manometry requires placement of a balloon catheter, correct positioning and occlusion testing, calibration and expertise in interpreting the pressure waveform, and it is uncomfortable in an awake, dyspnoeic patient wearing a tight-fitting mask, in whom catheter placement may itself precipitate intolerance. Few units outside research centres perform it routinely during NIV. Consequently, neither the tidal volume thresholds of 9 to 9.5 mL/kg of predicted body weight nor the 10 cmH_2_O oesophageal pressure criterion should be adopted into routine practice before prospective external validation in independent cohorts, ideally using non-invasive surrogates of effort that are feasible at scale.

Composite Scores

Six studies evaluated the HACOR score, a composite of heart rate, acidosis, consciousness, oxygenation and respiratory rate in which the respiratory items carry substantial weight (Table 3). In the derivation and validation study, 449 hypoxaemic patients formed the test cohort and 358 the validation cohort, with failure rates of 47.8% and 39.4%; measured one hour after initiation, the score achieved an area under the curve of 0.88, and a cut-off of 5 points retained good diagnostic accuracy across subgroups defined by diagnosis, age and severity, and across time points [21]. Applied to COPD, areas under the curve at one to two hours were 0.90, 0.89 and 0.71 in derivation, internal validation and external validation cohorts of 500, 323 and 395 patients, and 0.91, 0.96 and 0.83 for early failure specifically; among patients scoring above 5, failure occurred in 50.2%, and within this high-risk group early intubation within 48 hours was associated with lower hospital mortality (unadjusted odds ratio 0.15, 95% CI 0.05-0.39) [24]. In 148 non-COPD patients with acute-on-chronic hypercapnic failure, of whom only 19 (13%) failed, areas under the curve were 0.69 at initiation and 0.91, 0.91 and 0.94 after one to two, 12 and 24 hours, with optimal cut-offs falling from 7 points at initiation to 2 points at 24 hours [28].

External validation outside China was provided by a Spanish retrospective cohort of 2,749 NIV episodes in 2,711 consecutive patients, in which failure occurred in 963 episodes (35%). The score did not differ between success and failure before NIV but differed clearly at 1, 6, 12, 24 and 48 hours, with an area under the curve at one hour of 0.88 that exceeded 0.90 in the pneumonia and ARDS subgroups [26]. In 224 patients with moderate-to-severe ARDS and a PaO_2_/FiO_2_ of 150 mmHg or less, 125 (56%) failed NIV; patients whose score fell by at least one point after one to two hours had markedly lower failure (36% versus 72%, p < 0.01) and lower 28-day mortality (32% versus 47%, p = 0.04), and among intubated patients survivors had a shorter median interval from initiation to intubation than non-survivors (10 versus 22 hours) [29].

The score was subsequently updated to incorporate six baseline variables, namely pneumonia, cardiogenic pulmonary oedema, pulmonary ARDS, immunosuppression, septic shock and the Sequential Organ Failure Assessment score, using a training cohort of 1,451 patients and an external validation cohort of 728 patients from further hospitals in China and Turkey [30]. Measured at one to two hours, the updated score achieved areas under the curve of 0.85 (95% CI 0.84-0.87) and 0.78 (95% CI 0.75-0.81) in the training and validation cohorts, significantly better than the original score at every time point tested (all p < 0.01). Cut-offs of 7, 10.5 and 14 points corresponded to failure probabilities of 25%, 50% and 75%, and observed failure rates across the resulting strata were 12.4%, 38.2%, 67.1% and 83.7%. It is important to note what this improvement represents. Four of the six added variables, namely immunosuppression, septic shock, the Sequential Organ Failure Assessment score and the aetiological categories, are markers of systemic illness and case-mix rather than of respiratory function. The gain in discrimination therefore reflects, at least in part, the addition of baseline prognostic information about the patient's underlying condition rather than any improvement in the measurement of the respiratory response to NIV, and the updated instrument is best understood as a hybrid case-mix and response model rather than as a purely respiratory index.

Several limitations qualify the composite-score literature as a whole. Five of the six HACOR studies were conducted in China, and four originated from overlapping registries in a single city, so the apparent replication is narrower than the number of publications suggests, and independent external validation rests substantially on one Spanish retrospective cohort [26]. All are observational. Most importantly, incorporation bias is intrinsic to this instrument: oxygenation and respiratory rate are simultaneously items within the score and among the clinical criteria on which the treating team decides to intubate, so the predictor and the outcome are not independent. Discrimination falls appreciably on external validation, from 0.90 to 0.71 for the original score in COPD [24] and from 0.85 to 0.78 for the updated version [30], which is the expected pattern when a model is transported beyond its derivation setting.

Timing of Assessment

A consistent temporal pattern emerged across parameter domains and is summarised in Table 4. Pre-treatment values of every parameter examined discriminated only modestly: the ROX index before NIV achieved an area under the curve of 0.64 (95% CI 0.61-0.67) [31], the HACOR score at initiation achieved 0.69 in acute-on-chronic failure [28], and in the largest external validation the score before NIV did not differ at all between subsequent success and failure [26]. Discrimination improved substantially once NIV had been running for one to two hours and, for most indices, continued to improve at 12 and 24 hours [26,28,31]. The pattern is consistent across oxygenation [14,23], pH [15,25], tidal volume [20,23], inspiratory effort [27] and composite scores [21,26,28-30], and the most reasonable interpretation is that the response to treatment, rather than the pre-treatment state, carries most of the prognostic information.

This interpretation must be advanced with care. No included study was designed to compare assessment time points as its primary question; the temporal pattern is assembled from within-study comparisons reported secondarily, and the time points themselves were not standardised across studies. Protocols for NIV and thresholds for intubation differed between the contributing cohorts, and all were observational, so no causal inference can be drawn about the effect of assessing at one time point rather than another. Whether a single optimal reassessment time can be recommended is therefore not settled by this evidence. What can be said is that one to two hours is the earliest point at which discrimination becomes substantial across every domain examined, which makes it a defensible minimum standard for structured reassessment. Beyond that, the data suggest that timing may need to be individualised by underlying cause: in type II hypercapnic failure, pH corrects or fails to correct within the first one to two hours and late deterioration after 48 hours is a distinct phenomenon requiring continued vigilance [13,25], whereas in de novo hypoxaemic failure discrimination continues to improve out to 24 hours [26,31], implying that a single early assessment is necessary but not sufficient in that population. Establishing an optimal schedule would require a prospective study designed for that purpose, which does not yet exist.

Mortality and Intubation Timing

Most included studies reported mortality according to NIV outcome, and wherever reported, failure was associated with substantially worse survival. The endpoints are not equivalent and are therefore reported here with their timeframes rather than as a single figure: hospital mortality was 45.4% after failure compared with 16.1% after success in the international ARDS cohort [22]; hospital mortality was 92% compared with 3% among COPD patients experiencing late failure [25]; 28-day mortality was 32% in patients whose HACOR score improved at one to two hours compared with 47% in those whose score did not [29]; intensive care unit mortality was 46% among all intubated patients in a French cohort [18]; and 90-day mortality was independently associated with a tidal volume above 9 mL/kg of predicted body weight at one hour [23]. Direct comparison across studies is not appropriate, since intensive care unit, hospital, 28-day and 90-day mortality capture different periods of risk.

Three studies addressed the timing of intubation, and their populations differ substantially. Among high-risk COPD patients with a HACOR score above 5, intubation within 48 hours was associated with lower hospital mortality than later intubation [24]; among patients with moderate-to-severe ARDS, survivors were intubated a median of 12 hours earlier than non-survivors [29]; but in a French cohort of non-hypercapnic hypoxaemic patients, time to intubation showed no independent influence on intensive care unit mortality [18]. These are respectively a type II hypercapnic population, a severe ARDS population and a mixed de novo hypoxaemic population, in which the mechanisms of deterioration, the reversibility of the underlying process and the local thresholds for intubation all differ. The apparent disagreement between them may therefore reflect genuine differences between phenotypes rather than conflicting evidence about a single effect, and none of the three was designed to test the timing of intubation as an intervention.

Risk of Bias

**Figure 2:**
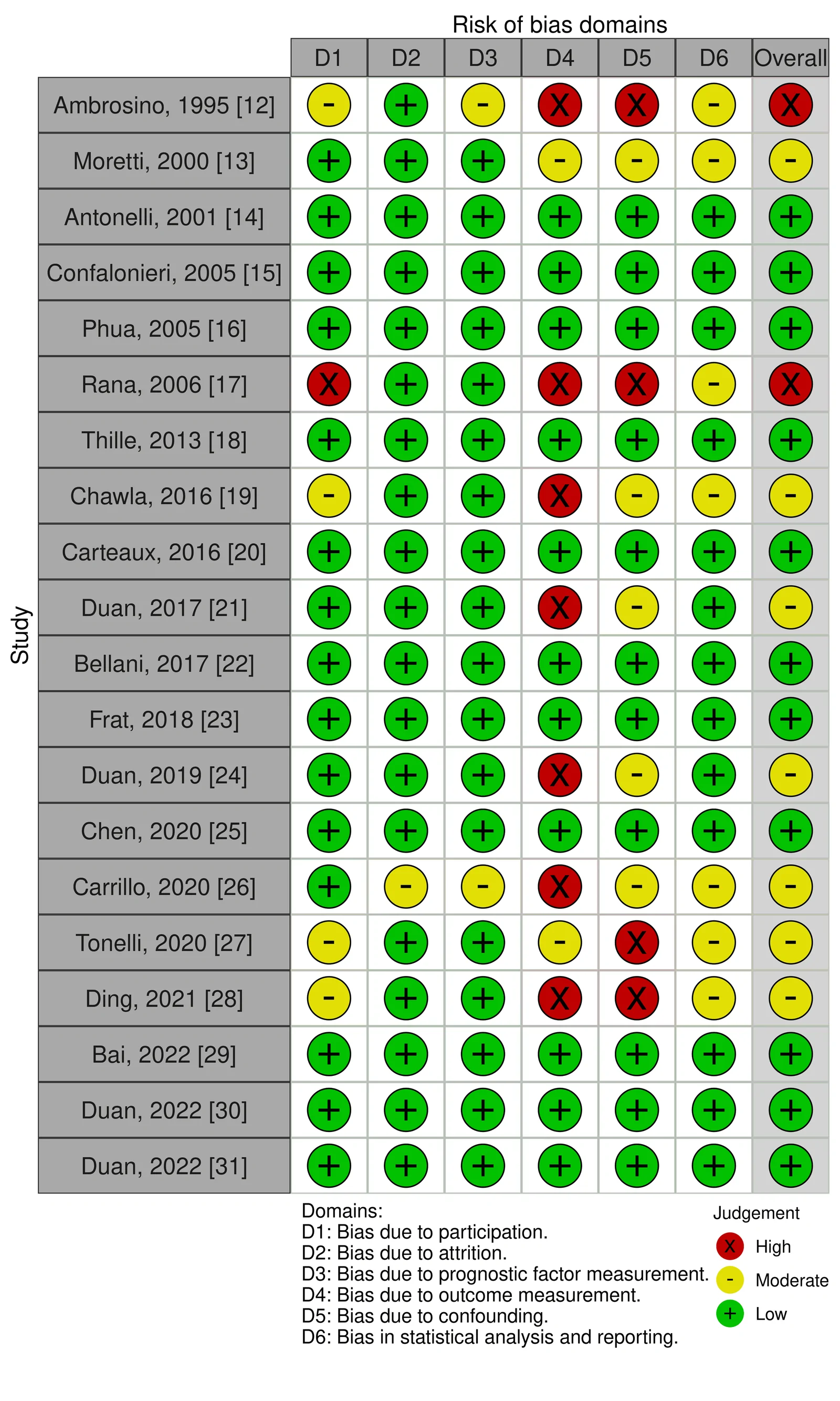
Risk-of-bias assessment using the Quality In Prognosis Studies (QUIPS) tool Studies included [12-31]

Judgements across the six QUIPS domains are presented in Figure 2. Eleven studies were rated at low overall risk of bias, seven at moderate risk and two at high risk [12,17]. Study participation was downgraded in five studies [12,17,19,27,28], attrition in one [26] and prognostic factor measurement in two [12,26], reflecting consecutive enrolment, complete in-hospital follow-up and the use of objective, routinely recorded measurements in most cohorts.

Outcome measurement was the most frequently downgraded domain, rated at high risk in seven studies [12,17,19,21,24,26,28] and at moderate risk in two [13,27]. This reflects unblinded, clinician-determined intubation and, in several of the composite-score cohorts, overlap between the predictor and the criteria used to decide on intubation. Confounding was rated at high risk in four studies in which the number of outcome events was small relative to the number of candidate predictors, or adjustment was limited [12,17,27,28], and statistical analysis and reporting at moderate risk in seven [12,13,17,19,26,27,28]. Calibration was reported in only a minority of the prediction-model studies.

These judgements bear directly on how far the conclusions of this review can be trusted, and they do not affect all findings equally. The domains most compromised are precisely those carrying the headline results: outcome measurement was rated high risk in four of the six HACOR studies and confounding was rated high risk in the single inspiratory-effort study, so the two most striking accuracy figures in the review, the areas under the curve of 0.85 to 0.94 for composite scores and of 0.97 for oesophageal pressure swing, are the estimates most likely to be optimistic. By contrast, the finding that respiratory rate loses predictive value under NIV rests on the one study rated at low risk across all six domains [23] and is correspondingly more secure. The overall effect is that the direction of the review's conclusions is well supported, while the absolute magnitude of the reported discrimination should be regarded as an upper bound. This reasoning is formalised in the certainty ratings below, and it is the reason no domain was rated high certainty. Finally, because no meta-analysis was undertaken, publication bias could not be assessed by funnel plot or asymmetry testing; small prognostic studies reporting positive findings are more likely to be published, so a degree of upward bias in the published thresholds cannot be excluded.

Certainty of Evidence

Certainty ratings by parameter domain are reported in the final column of Table 4. Certainty was moderate for oxygenation indices, for acid-base status in type II failure, and for composite scores, each supported by consistent findings across multiple cohorts but downgraded once for risk of bias, principally unblinded outcome ascertainment and incorporation bias. Certainty was low for respiratory rate, downgraded for inconsistency because the direction of association reversed once NIV was applied, and low for tidal volume and inspiratory effort, downgraded for imprecision and indirectness given the small number of independent cohorts and the single 30-patient study underpinning the effort data. No domain reached high certainty, because no included study was designed to test whether acting on a threshold changes patient outcome.

Discussion

Main Findings

This systematic review of 20 primary studies, comprising approximately 11,700 patient-episodes accumulated over 27 years in 10 countries, supports a coherent and clinically useful message: the predictive value of respiratory parameters for NIV failure appears to be dynamic rather than static. Almost every parameter examined discriminated poorly or only modestly when measured before NIV was started, and discriminated well when measured one to two hours afterwards. The HACOR score moved from an area under the curve of 0.69 at initiation to 0.91 at one to two hours in acute-on-chronic hypercapnic failure [28]; in the largest external validation cohort the score before NIV did not distinguish subsequent success from failure at all, whereas at one hour it achieved 0.88 [26]; and the ROX index followed the same trajectory [31]. The most plausible interpretation is that what is measured at one hour reflects the responsiveness of the patient to positive pressure rather than the severity of the illness alone, and that it is responsiveness that carries much of the prognostic information. The bedside consequence is that the useful question is not only whether a patient is sick enough to warrant intubation before NIV is attempted, but whether the physiological derangement has improved after a short and closely supervised trial.

Comparative Value of Individual Parameters

Within this framework, the individual parameters are not interchangeable. Oxygenation indices were the most consistent single predictors across the case-mixes examined, with independent adjusted odds ratios of 2.51 at one hour [14] and 4.26 at one hour for a lower threshold [23], and with a graded relationship between ARDS severity category and failure rate observed both in a single French centre and across 50 countries [18,22]. Respiratory rate, by contrast, was the weakest stand-alone predictor once NIV had been applied. The clearest demonstration came from the post hoc analysis of a randomised comparison of oxygenation strategies, in which a respiratory rate of 30 breaths per minute or more predicted intubation under standard oxygen but lost its predictive value under NIV or high-flow nasal oxygen [23]. Pressure support directly unloads the respiratory muscles and reduces respiratory frequency without necessarily improving alveolar recruitment, shunt or the underlying inflammatory process, so a normalised respiratory rate under NIV may be falsely reassuring. This is among the most useful practical cautions to emerge from the review, because respiratory rate is the parameter most commonly used informally at the bedside to judge whether a patient is settling on NIV. It rests, however, on a single study, and the trajectory of respiratory rate as distinct from its absolute value has not been tested.

The studies of tidal volume and inspiratory effort suggest a mechanistic link that may explain why oxygenation-based prediction works and why respiratory rate does not. In de novo hypoxaemic failure, no patient in one prospective cohort maintained a mean expired tidal volume below 6 mL/kg of predicted body weight during NIV, and a tidal volume above 9.5 mL/kg predicted failure in moderate-to-severe hypoxaemia [20]; a threshold of 9 mL/kg was independently confirmed at one hour in a separate multicentre dataset, where it also predicted 90-day mortality [23]. Direct measurement of effort by oesophageal manometry then showed that patients destined to fail were distinguished at two hours not by their gas exchange but by an unremitting inspiratory effort [27], although that finding comes from 30 patients and awaits validation. Together these observations are consistent with patient self-inflicted lung injury operating during NIV rather than remaining a theoretical concern, and they suggest that the phenotype at risk is the patient whose respiratory drive remains high, who continues to generate large tidal volumes, and whose oxygenation consequently fails to improve. Supporting evidence from outside this review points the same way: in critically ill patients with COVID-19, high respiratory drive and excessive respiratory effort predicted relapse of respiratory failure [32], and a randomised trial showed that interface choice, which alters the transmission of airway pressure and the achievable tidal volume, changes intubation rates in ARDS [33].

Comparison With Existing Literature

Comparison with the wider literature is instructive in two directions. It should be noted that the studies cited in this section and the next were not eligible for inclusion in the review, either because they evaluated high-flow nasal oxygen rather than NIV, because they were systematic reviews rather than primary studies, or because they did not analyse a respiratory parameter in relation to NIV failure; they are cited as supporting context only, and all evidence contributing to the findings of this review is drawn from references 12 to 31. First, the trajectory of the ROX index under NIV closely parallels its behaviour under high-flow nasal oxygen, where the original validation cohort demonstrated an increase in discrimination from 0.679 at two hours to 0.759 at 12 hours and identified a value of 4.88 or above as marking likely success [9]; subsequent meta-analyses have confirmed its usefulness for high-flow nasal oxygen while noting substantial between-study heterogeneity in thresholds [34,35]. That the same index behaves similarly under a different mode of support is consistent with its capturing a general property of treatment responsiveness rather than a device-specific effect. Second, the finding that oxygenation-anchored composites outperform any single variable is consistent with the broader prognostic literature in critical care, and with the observation that adding baseline case-mix information to the HACOR score improved discrimination at every time point tested [30], although, as noted above, much of that added information is non-respiratory. It is notable that even the updated score achieved an area under the curve of only 0.78 on external validation, appreciably lower than the 0.85 obtained in the training cohort. This degree of optimism is typical of prediction models and argues against transporting locally derived thresholds into a new setting without local validation.

Gaps in the Evidence Base

The first gap concerns phenotypic and geographical concentration. Seven of the 20 studies originated from a small number of overlapping registries in Chongqing, China [21,24,25,28-31], which means that the apparent weight of evidence supporting the HACOR score is smaller than a simple count of studies suggests, and that its external generalisability rests substantially on a single Spanish cohort [26]. Just as important is the narrowness of the clinical phenotypes represented. The evidence base is drawn almost entirely from three populations: COPD exacerbation, de novo hypoxaemic respiratory failure and ARDS. Cardiogenic pulmonary oedema, one of the two indications for which NIV has the strongest randomised support, is not represented by any included study as a discrete population, nor are obesity-related hypoventilation, neuromuscular respiratory failure, post-operative respiratory failure, or immunocompromised patients, in whom the risk-benefit balance of NIV is particularly contested. Only one included study examined an ARDS population outside Europe, North America and East Asia [19], despite pneumonia being among the diagnoses most strongly associated with failure in both older and more recent cohorts [14,36]. Supporting work published outside this review has suggested that the underlying aetiology of ARDS modifies failure risk independently of oxygenation [37], a dimension that none of the parameter-based tools reviewed here captures. The type I to type II imbalance compounds this: hypoxaemic patients contributed roughly three-quarters of the evidence base, so the acid-base findings that dominate management of type II failure rest on a proportionately smaller body of data.

The second gap concerns the outcome itself. NIV failure, as operationalised across these studies, is a composite of a physiological event and a clinician decision, and the two cannot be fully disentangled in observational data. Outcome ascertainment depended on a decision to intubate that was not blinded to the parameter under evaluation, and in several of the composite-score cohorts the parameter itself, or a close correlate, contributed to that decision. This incorporation bias inflates apparent discrimination in a way that cannot be corrected analytically, and it was the reason outcome measurement was rated at high risk of bias in seven studies. A further consequence is that failure rates cannot be compared meaningfully across studies applying different thresholds for intubation; the more than fivefold range in reported failure, from 13% [28] to 70% [17], reflects case-mix and local practice at least as much as any biological difference. Related to this, NIV intolerance is a distinct and common pathway to failure, driven by discomfort, agitation and interface problems rather than by gas exchange, and it is not captured by any of the parameters reviewed here [38].

The third gap is methodological. Discrimination was reported in most of the prediction studies, but calibration, the agreement between predicted and observed risk, was reported in only a minority, despite being the property that determines whether a stated threshold means the same thing in a new population. Decision-curve or net-benefit analyses were absent altogether. Most importantly, no included study was an impact study: none randomised patients to management guided by a respiratory-parameter threshold versus usual care, and so none can demonstrate that acting on these predictions improves outcome. This is the principal reason no parameter domain reached high certainty. The observational signals that early intubation in high-risk patients is associated with lower mortality [24] and that survivors are intubated earlier than non-survivors [29] are encouraging but vulnerable to confounding by indication, and they are not reproduced in a cohort where time to intubation had no independent influence on mortality [18]. A fourth gap concerns reporting of the intervention itself: ventilator settings were described too inconsistently across the included studies to permit any aggregate statement about the pressures or oxygen fractions under which these predictors were derived, which limits precise translation to the bedside.

Implications for Clinical Practice

NIV for ARF is best conceived as a time-limited diagnostic as well as therapeutic trial, with a structured reassessment at one to two hours that includes arterial blood gas analysis, respiratory rate, delivered or expired tidal volume where the ventilator permits it to be measured, and a composite score. Deterioration or failure to improve in oxygenation, persistence of acidosis in hypercapnic failure, an expired tidal volume persistently above 9 mL/kg of predicted body weight, or a composite score that has not fallen should each prompt intensified monitoring and preparation for intubation rather than continued observation alone. A normalised respiratory rate in the presence of unchanged oxygenation should not by itself be accepted as evidence of response. Where oesophageal manometry is available, failure of the pressure swing to fall by at least 10 cmH_2_O at two hours was the most discriminating single measurement identified in this review, but it derives from one 30-patient pilot study, was rated low certainty, and is not yet ready for routine use. These inferences are drawn from observational evidence of moderate certainty at best, and are offered as a structured prompt to reassessment rather than as validated decision rules. Because thresholds derived in one health system have transported imperfectly to another, and because almost all of this evidence comes from intensive care units, units adopting any of these tools, particularly outside critical care, should audit their own observed failure rates against predicted risk before allowing the tool to influence decisions.

Future Research

Future research should prioritise three objectives. Prospective external validation of the updated composite scores in populations outside the derivation regions, with explicit reporting of calibration as well as discrimination, would establish whether the published thresholds are transportable, and should extend to the phenotypes currently unrepresented, particularly cardiogenic pulmonary oedema and immunocompromised patients. Studies incorporating direct measures of effort and drive would test whether the accuracy observed with oesophageal manometry can be reproduced at scale; several emerging approaches deserve evaluation, including non-invasive surrogates of effort such as diaphragmatic ultrasonography and airway occlusion pressure, regional ventilation monitoring by electrical impedance tomography, continuous capnography, and machine-learning models that exploit the full time series of bedside physiological data rather than isolated time points. Future studies should also report ventilator settings, interfaces and monitoring modalities in standardised detail, and should analyse the trajectory of each parameter rather than single values. Above all, a randomised impact trial comparing protocolised, threshold-driven escalation with usual clinical judgement is required to determine whether accurate prediction translates into patient benefit. Until that trial is done, the fair summary of this literature is that respiratory parameters measured shortly after NIV initiation identify, with useful and reasonably reproducible accuracy, the patients in whom NIV is not working, and that recognising those patients early is plausibly, but not yet demonstrably, better than recognising them late.

Limitations

Several limitations apply. The review was not registered prospectively with the International Prospective Register of Systematic Reviews (PROSPERO), which reduces protection against selective reporting, and formal inter-rater agreement statistics were not calculated at screening or risk-of-bias assessment. Meta-analysis was not feasible because of substantial clinical and methodological heterogeneity: the included studies differed in ARF aetiology and physiological type, NIV interfaces and ventilator settings, thresholds and timing of measurement, definitions of failure, criteria for intubation, clinician experience and healthcare system, and the balance of intensive care versus ward settings. Consequently, no pooled estimates are available, and publication bias could not be assessed formally. Cohort overlap is a further constraint: seven of the 20 studies drew on overlapping registries in a single Chinese city, only one duplication could be quantified, and the true number of unique patients is therefore fewer than the approximately 11,700 patient-episodes reported and cannot be established precisely. The evidence is almost entirely observational, precluding causal inference, and incorporation bias is likely to have inflated reported discrimination; no parameter domain reached high certainty. The evidence base is also weighted towards type I hypoxaemic failure, which contributed about three-quarters of patient-episodes, and towards three phenotypes only, so cardiogenic pulmonary oedema, immunocompromised and post-operative patients are unrepresented. Baseline demographic and severity data could not be aggregated because severity was reported using three different instruments; ventilator settings and monitoring were reported too incompletely to permit aggregation; no included study evaluated the helmet interface or continuous capnography; and inspiratory effort was measured directly in only one 30-patient study.

## Conclusions

Respiratory parameters predict NIV failure most usefully when interpreted dynamically. Pre-treatment values carry limited prognostic information, whereas values obtained one to two hours after initiation, and the direction of their change, discriminate considerably better. Oxygenation indices are the most consistent single predictors; composite scores incorporating oxygenation, acidosis, consciousness, heart rate and respiratory rate perform better still, reaching areas under the curve of 0.85 to 0.94 at one to two hours in derivation cohorts though falling to 0.71 to 0.78 on external validation; and in de novo hypoxaemic failure a persistently high expired tidal volume or an unrelieved inspiratory effort identifies patients at high risk. Respiratory rate alone is not a dependable indicator of response once NIV has been applied. A trial of NIV should therefore be structured and time-limited, with physiological reassessment at one to two hours and an explicit decision point rather than open-ended observation. These conclusions should be read with their limitations in view: the evidence is almost entirely observational, clinical and methodological heterogeneity was substantial, incorporation bias was common because the predictors also inform the decision to intubate, several large cohorts overlapped, and external validation remains incomplete. Confidence in this body of evidence is therefore moderate rather than high, and prospective external validation with calibration reporting, together with a randomised trial of threshold-guided escalation, is needed before accurate prediction can be assumed to improve outcomes.

## Appendices

Full electronic search strategies

All four databases were searched from inception to 25 June 2026. No language, geographical or publication-status limits were applied at the search stage. Yields correspond to the counts reported in Figure 1.

**Table 5:** Search strategy

Database	Search string	Records retrieved
PubMed/MEDLINE	("noninvasive ventilation"[MeSH] OR "non-invasive ventilation"[tiab] OR "noninvasive ventilation"[tiab] OR NIV[tiab] OR NPPV[tiab] OR NIPPV[tiab] OR "bilevel positive airway pressure"[tiab] OR BiPAP[tiab] OR "continuous positive airway pressure"[MeSH]) AND ("treatment failure"[MeSH] OR failure[tiab] OR "intubation, intratracheal"[MeSH] OR intubation[tiab] OR escalation[tiab]) AND ("respiratory rate"[tiab] OR "tidal volume"[MeSH] OR "tidal volume"[tiab] OR "blood gas analysis"[MeSH] OR "PaO2/FiO2"[tiab] OR "P/F ratio"[tiab] OR "SpO2/FiO2"[tiab] OR "ROX index"[tiab] OR HACOR[tiab] OR pH[tiab] OR PaCO2[tiab] OR "esophageal pressure"[tiab] OR "inspiratory effort"[tiab] OR predict*[tiab]) AND ("respiratory insufficiency"[MeSH] OR "acute respiratory failure"[tiab] OR ARDS[tiab] OR "respiratory distress syndrome"[tiab])	257
Scopus	TITLE-ABS-KEY(("non-invasive ventilation" OR "noninvasive ventilation" OR NIV OR NPPV OR NIPPV OR "bilevel positive airway pressure" OR BiPAP OR "continuous positive airway pressure" OR CPAP) AND (failure OR intubation OR escalation OR "treatment failure") AND ("respiratory rate" OR "tidal volume" OR "blood gas" OR "PaO2/FiO2" OR "P/F ratio" OR "SpO2/FiO2" OR "ROX index" OR HACOR OR pH OR PaCO2 OR "esophageal pressure" OR "inspiratory effort" OR predict*) AND ("acute respiratory failure" OR "respiratory insufficiency" OR ARDS OR "respiratory distress syndrome"))	82
Embase (Elsevier)	('noninvasive ventilation'/exp OR 'non-invasive ventilation':ti,ab OR NIV:ti,ab OR NPPV:ti,ab OR NIPPV:ti,ab OR 'bilevel positive airway pressure':ti,ab OR BiPAP:ti,ab OR 'positive end expiratory pressure'/exp) AND ('treatment failure'/exp OR failure:ti,ab OR 'endotracheal intubation'/exp OR intubation:ti,ab) AND ('respiratory rate':ti,ab OR 'tidal volume'/exp OR 'arterial gas'/exp OR 'PaO2/FiO2':ti,ab OR 'SpO2/FiO2':ti,ab OR 'ROX index':ti,ab OR HACOR:ti,ab OR pH:ti,ab OR PaCO2:ti,ab OR 'esophageal pressure':ti,ab OR 'inspiratory effort':ti,ab OR predict*:ti,ab) AND ('respiratory failure'/exp OR 'acute respiratory failure':ti,ab OR ARDS:ti,ab)	74
Web of Science Core Collection	TS=(("non-invasive ventilation" OR "noninvasive ventilation" OR NIV OR NPPV OR NIPPV OR "bilevel positive airway pressure" OR BiPAP OR "continuous positive airway pressure") AND (failure OR intubation OR escalation) AND ("respiratory rate" OR "tidal volume" OR "blood gas" OR "PaO2/FiO2" OR "SpO2/FiO2" OR "ROX index" OR HACOR OR pH OR PaCO2 OR "esophageal pressure" OR "inspiratory effort" OR predict*) AND ("acute respiratory failure" OR "respiratory insufficiency" OR ARDS OR "respiratory distress syndrome"))	97
Total records identified		510
